# A Quantitative Theory of Human Color Choices

**DOI:** 10.1371/journal.pone.0055986

**Published:** 2013-02-11

**Authors:** Natalia L. Komarova, Kimberly A. Jameson

**Affiliations:** 1 Department of Mathematics, University of California Irvine, Irvine, California, United States of America; 2 Institute for Mathematical and Behavioral Sciences, University of California Irvine, Irvine, California, United States of America; University of Muenster, Germany

## Abstract

The system for colorimetry adopted by the *Commission Internationale de l’Eclairage* (CIE) in 1931, along with its subsequent improvements, represents a family of light mixture models that has served well for many decades for stimulus specification and reproduction when highly controlled color standards are important. Still, with regard to *color appearance* many perceptual and cognitive factors are known to contribute to color similarity, and, in general, to all cognitive judgments of color. Using experimentally obtained odd-one-out triad similarity judgments from 52 observers, we demonstrate that CIE-based models can explain a good portion (but not all) of the color similarity data. Color difference quantified by CIELAB ΔE explained behavior at levels of 81% (across all colors), 79% (across red colors), and 66% (across blue colors). We show that the unexplained variation cannot be ascribed to inter- or intra-individual variations among the observers, and points to the presence of additional factors shared by the majority of responders. Based on this, we create a quantitative model of a lexicographic semiorder type, which shows how different perceptual and cognitive influences can trade-off when making color similarity judgments. We show that by incorporating additional influences related to categorical and lightness and saturation factors, the model explains more of the triad similarity behavior, namely, 91% (all colors), 90% (reds), and 87% (blues). We conclude that distance in a CIE model is but the first of several layers in a hierarchy of higher-order cognitive influences that shape color triad choices. We further discuss additional mitigating influences outside the scope of CIE modeling, which can be incorporated in this framework, including well-known influences from language, stimulus set effects, and color preference bias. We also discuss universal and cultural aspects of the model as well as non-uniformity of the color space with respect to different cultural biases.

## Introduction

Humans constantly experience environmental color and interact with color appearance in everyday life. Because the perceptual processing of color arose as an adaptation through biological and cultural evolution, scientific analyses of human color processing must involve many factors, including physical properties of color stimuli, the processing of physical reflectance features by the human visual system, and higher-order cognitive processing of color in human visual cortex. On the other hand, modern scientific study of human interactions with color stimuli grew largely out of early engineering and technological applications where color specification and reproduction in industry are of crucial importance. As a result, a key emphasis in the early days of developing photograph and display technology was to provide methods to precisely define color appearance, and to do so in ways that were adequate for reproducing color across different formats (for example, to make sure that the exact appearance of the yellow of Kodak film packaging is maintained in all its marketed forms).

A consequence of this applied emphasis was the founding of the international authority on light, illumination and color, established in 1913, known as the *Commission Internationale de l’Eclairage* (or *CIE*) which led to a 1922 report on colorimetry by the Optical Society of America, and subsequently the formalization of the CIE 1931 XYZ color specification and the 1931 CIE 2-degree standard observer color space and human color matching functions, as well as a series of subsequent standard observer color space refinements [1], [2].

Given its originally intended use, it is amazing that CIE systems have served so well as models of complex color appearance computations. Still, it is well recognized that other perceptual and cognitive factors also contribute to color similarity, and, in general, to all cognitive judgments of color. We know, for example, that individual variation in visual processing features result in nonuniform color perception across observers [3], [4] and that those CIE models most widely used in the color perception literature (i.e., CIELAB and CIELUV) are not entirely perceptually uniform and they do not take into consideration many factors contributing to individual differences in color perception [5], [6]. Although perceptually uniform spacing has not been forthcoming, CIELAB distances are still used in empirical investigations to choose stimuli across color space that, for example, are quantified to appear equal in perceptual qualities based on the CIELAB ΔE distance metric. Moreover, cultural and linguistic biases can significantly alter color similarity judgments [7], [8]. Thus, various cognitive factors must be involved in color similarity judgments, but aside from technical extensions of CIELAB (e.g., [9], [10]) there is no clear principled approach for incorporating these factors into the existing modeling framework given by the CIE. The present paper attempts to address this challenge by incorporating cognitive modeling features to extend the potential of CIE systems as the basis for color similarity relations.

In this paper we investigate whether CIE models are a good predictor of human choice behavior in color similarity judgments. We use results from a color triad task. Color triad tasks require observers to choose from three-color samples the singleton that does not form a natural grouping, or “belong with”, the other two of samples provided. Such tasks have been used with the aim of understanding biological, psychophysical and cognitive constraints that guide the human responders in their choices [11], [12], [13]. Color triad judgments provide an approach to bridge the gap between color specification models (i.e., tristimulus light mixture and color order systems) and the modeling of cognitive color relations. Using color similarity choice data we discuss the appropriateness and utility of a CIE-based approach when other forms of cognitive influence are involved in color triad choice or other cognitive color judgment data. We analyze triad data from subjects who provided color similarity judgments both across color categories (i.e., reds, yellows, greens, etc.) referred to below as “global colors”, and within color categories (i.e., judged similarity among samples of red colors, or judged similarity among blue colors) referred to as “local red” and “local blue” color sets. Our findings support the use of some features of CIE models as a cognitive color appearance model when the model is extended to include additional color space features that figure into the cognitive color judgments. To illustrate, we increase the complexity of the model used to predict respondents’ triad choice data, for example by modeling influences of language-based categorical constraints expected to influence color judgments, and further analyze the fit to the triad choice behavior. Our results suggest that CIE distance is but the first layer in a hierarchy of influences that shape triad choices. Other mitigating influences come from language, stimulus set effects, and color preference bias.

## Results

Let us represent each color triad as a triangle in a three-dimensional CIE space. The likelihood for a given stimulus in one such triad to be chosen as an odd-one-out is, in the simplest null-model, dictated by the geometry of the triangle [7], [8]. The “null-model” assumes this probability to be proportional to the inverse of length of the opposing edge of the triangle (raised to a power, 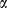). For example, for a triangle of colors with vertices A, B, and C, the probabilities for colors A, B and C to be chosen are given by
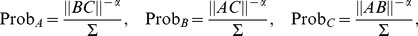
(1)where 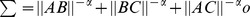. Applied to experimental triad items, this model produces, for each color stimulus, its probability to be chosen, which can be compared to the empirically observed probabilities given by responders’ choices. There are different ways this comparison can be implemented. We can vary: (a) the method of distance measurement, using a ΔE color difference index based on CIELAB, CIE94, or CIEDE2000 [2]; (b) the way the “winning” stimulus is chosen from the multitude of responses, the majority or the consensus; (c) color similarity relations can be based on choice data from all participants, from participants grouped by their color vision profile, or on data from individual observers. We varied all these factors to obtain the most comprehensive picture of the validity of the model (see Text S1). A convenient measure of the CIE model’s performance is the number of mismatches that it produces compared to the experimental observations, that is, the number of triads where CIE distances predict a different odd-one-out stimulus compared to that chosen by experimental participants. For the CIELAB distance measure, the majority calculation of the winning response and the full set of participants, the number of mismatches is 13, 15, and 24 for the global, red, and blue conditions respectively. That is, distances of the CIELAB model alone can explain from about 66% (the blue condition) to 81% (the global condition) of the data. Beyond color differences implied by CIE distance, what are some of the other factors possibly contributing to participants’ triad choice behavior?

There are three possible sources of variation that can contribute to mismatches between the data and the CIE model. (1) *Intra-individual variation* arises as a degree of test-retest inconsistencies of the observers, which are random chance events, and can be a reflection of individual behavior, such as sloppiness in completing the test. (2) *Inter-individual variation* can reflect (a) inhomogeneities of the observers, such as the presence of a small number of dichromats, (b) individual cognitive factors such as personal color preferences or linguistic influences relevant in subsets of participants. (3) *Systematic variation,* or patterns of deviation from the CIE model that are frequent in a large portion of the participants, which can arise from (a) inconsistencies in the CIE description of perceptual space, or (b) systematic cognitive factors such as variation in the assignment of shared color meaning or “conventions”. Inter- and intra-individual variations have previously been quantified and compared [14], [15]. The present model allows us to naturally incorporate such variations and study their properties (for example, the parameter 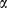 in equation (1) measures the amount if intra-individual variation, see Text S1). The three types of variation mentioned above behave differently as one varies the number of participants considered. By taking subsets of the responders we were able to show that the variation of type (1) or (2) alone cannot explain the existing difference between the null-model and the observed experimental results (see Text S1). Therefore, we conclude that there must exist some systematic source of difference between the null-model and the observations, common to the majority of the participants.

In order to improve the null-model, we propose that in addition to the distance considerations provided by the representation of colors in CIE space, there are other perceptual-cognitive factors that contribute to individual odd-one-out color similarity choices. Some of these factors may be widely used (such as a warm-cool distinction described below), whereas others may be more dependent on pragmatic uses of color or even culturally specific uses that vary across groups of individuals. As shown in analyses that follow, such factors can trade-off perceptually salient features for pragmatic features in a manner that resembles choice behavior interpreted using *lexicographic semi-order* modeling [16], an analogy that is detailed in the discussion section below.

One identifiable factor underlying choice behavior for color similarity that goes beyond the CIE distance model is a factor we refer to as “categorical considerations”. Categorical considerations permit the natural partitioning of the color stimuli studied into subsets ‘*A’* and ‘*not A’*. Such distinctions can be based on idiosyncracies of individual perceptual salience [17], [18], culturally relevant color utility [7], [19], [20], environmental signaling, [21] or any other feature on which a set of colors could be partitioned [22]. For example, categorical considerations of color similarity can be shaped by how color meaning is denoted by a society’s color language. The cross-cultural color-naming literature has proposed that color-triad choices are influenced by language category biases. That is, beyond color-based similarity features of stimuli, even in tasks that are strictly perceptual in nature odd-one-out choices may be influenced by where in color space a participant’s color lexicon draws a categorical distinction ([23], [24], [25], but also see [26] for an alternative view).

**Figure 1:**
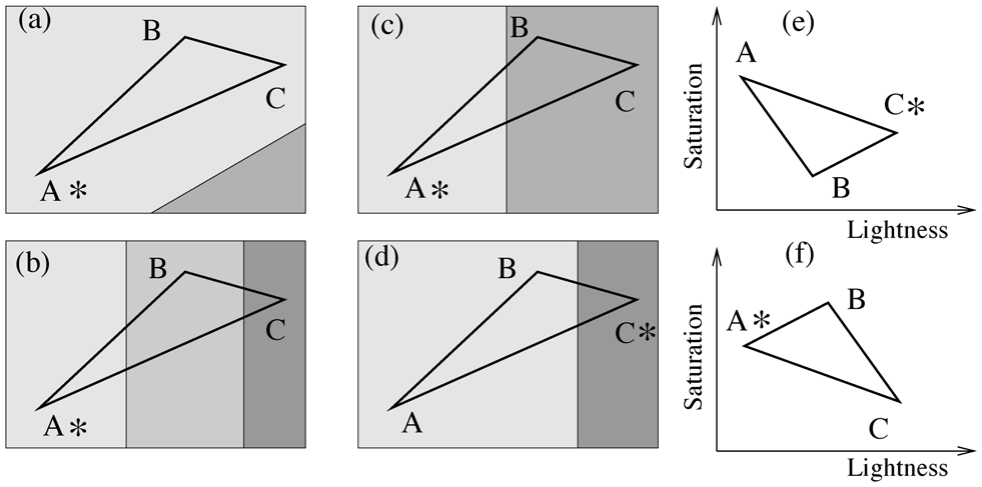
The category (a–d) and lightness-saturation (e,f) biases in triad choices. (a–d): A triad (A,B,C) is represented as a triangle in a CIE space. In the absence of categorical and other biases, stimulus A is the most likely choice. In the presence of categorical biases, the choice might shift. Different categories are denoted by different background shades, and the most likely choice is marked by a star. (a) All stimuli belong to the same category; A remains the most likely choice. (b) All three stimuli belong to different categories; the choice remains A.(c) Stimulus A belongs to a different category than B and C; the choice remains A. (d) Stimulus C belongs to a different category from A and B; the choice might shift from A to C. (e,f): The same triad projected into the lightness-saturation space (a schematic). (e) Stimulus A is the darkest and most saturated color; the choice might shift from A to C. (f) Stimulus A is not simultaneously the darkest and most saturated color; the choice remains A



Here categorical considerations are given a quantitative implementation. We propose that the probability to be chosen defined by equation (1) involves a correction factor based on category differences. Consider the example of Figure 1(a–d), where a triad is represented schematically as a triangle in a CIE space. In the absence of categorical biases, stimulus A in this triad would be the most likely choice. Next imagine there is a “partitioning” of color space that separates the stimuli into different categories. These categories are marked in Figure 1 by different background shading. If stimulus C belongs to a different category compared to the other two stimuli, there is a chance that C will become a more likely choice than A (Figure 1(d)). Mathematically, this is expressed by the formula

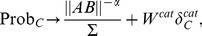
(2)which contains an additive term that modifies the probability that stimulus C is chosen. Probabilities of choosing the other two stimuli, A and B, are modified similarly, and then the three probability expressions are normalized such that they sum up to one. In equation (2) 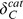 denotes the category indicator, which informs us whether category considerations play a role in this particular triad, for this stimulus C. In the example of Figure 1(d) where stimulus C belongs to a different category from stimuli A and B, we have 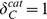. If however stimulus C belongs to the same category as any other stimulus in the given triad, then we set 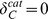 for this triad. In particular, if all three stimuli belong to the same category (Figure 1(a)), or if they belong to three different categories (Figure 1(c)), then all three stimuli have their respective 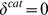, and category considerations are not expected to influence individual’s color similarity judgments. In the case depicted in Figure 1(b), we have 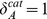, that is, category considerations strengthen the choice of stimulus A as the odd-one-out and essentially do not modify the prediction of the original model (1). In a given triad, only one stimulus can have a nonzero category indicator. The weight coefficient, 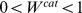, measures the relative importance of category biases compared to distance biases.

**Figure 2:**
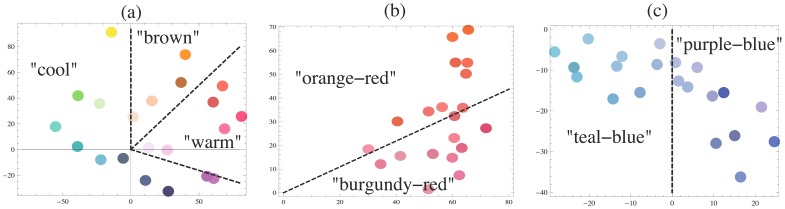
Optimal category choices in the three experimental conditions, global (a), red (b) and blue (c). The 21 stimuli in each condition are plotted on the a*b* plane (please note the scale difference among the three conditions). The color stimuli are presented by colored dots that approximate the colors of the stimuli. The categories are separated by radial dashed lines.

In the present analyses categories are based on considerations of hue and warm-cool biases (see Figure 2). In the two local conditions, two categories were considered, as shown in Figure 2 by radial dashed lines in the a*b* plane of CIELAB (1976) space [1]. The particular choice of the location of the dividing lines, as well as the numerical value of parameter *W^cat^*, was obtained as part of the optimization problem (see Text S1). In the red condition, the two categories that resulted in the best match of the model with the observed data, empirically correspond to “orange-red” and “burgundy-red” biases. In the blue category, we have “teal-blue” and “purple-blue” biases. In the case of the global condition (Figure 2(a)) we find not two but three different categories termed as “warm”, “cool” and “brown”. As an aside, the emergence of a separate “brown” factor, or category, in these data is not surprising since browns differ from other colored light mixtures in that they exist as “relational colors” that are experienced in the context of a brighter surrounding field, and in CIELAB or CIELUV spaces correspond to “orange” color space coordinates [1], implying that “brown” light mixtures are absent from those models. Similarity judgments on “relational” colors like brown are possible in these data because surrounding contrast was supplied by the gray background used in our triad stimulus configuration [27].

When we apply this model to the data at hand, we observe that the category correction helps improve the performance of the model considerably, especially in the global and red conditions. The least number of mismatches that this model produces is 8, 7 and 14 for the global, red, and blue conditions respectively, see Table 1. We conclude that category constraints and distances are not the whole picture, which is especially apparent in the blue condition. Other factors must be at play.

**Table 1:** The number of mismatches produced by different models (equations (1), (2), and (3), as well as the null model and lightness-saturation bias) for the global, red, and blue conditions.

	Global	Red	Blue
Null, eq.(1)	13	15	24
Null+category bias, eq.(2)	8	7	14
Null+category+ls bias, eq.(3)	6	7	9
Null+ls bias	12	15	11

In order to find the source of the remaining variation, we explored the hypothesis that alternative dimensional differences can influence participants’ choices. We notice a common pattern in many of the mismatches in the blue condition: the CIE prediction of the odd-one-out stimulus corresponds to the color with the lowest lightness coordinate and the highest saturation coordinate compared to the other two stimuli in the triad, see Figure 1(b). We make a hypothesis that people prefer *not* to choose the darkest and the most saturated color as the odd-one-out.

This leads to the following correction to model (2):
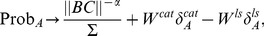
(3)where the superscript “*ls*” refers to lightness-saturation bias, 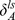 is the lightness-saturation indicator, and 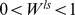 is the weight coefficient measuring the relative importance of the lightness-saturation bias (determined by a fitting procedure, see Text S1). As in equation (2), the other two probability values are modified similarly, and then normalized to make sure that they sum up to 1. In equation (3), the indicator 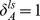 only if stimulus *A* is the darkest and most saturated color of the three; it is zero otherwise, see Figure 1(e, f). In the Figure, we presented a possible projection of the triad *ABC* onto the lightness-saturation space. If, as in Figure 1(e), stimulus *A* happens to be the darkest and the most saturated color, then its lightness-saturated bias will be set to one, and it may lead to a different choice of the odd-one-out color. In other cases, such as the one illustrated in Figure 1(f), the lightness-saturation bias of stimulus *A* will be zero. We fitted the model in equation (3), and the best-fitting model reduces the number of mismatches to 6, 7 and 9 for the global, red and blue conditions respectively. Interestingly, in the global and red conditions, lightness-saturation bias does not make much of a difference. When applied together with the category bias, or alone (see the last row of Table 1), it does not influence the number of mismatches significantly. In the blue condition however it plays an important role. When applied together with the category bias, it reduces the number of mismatches from 14 to 9, and when applied without the category bias, it reduces the number of mismatches from 24 to 11.

To summarize, the most comprehensive model (3) can explain 91% of the data for the global, 90% for the red, and 87% for the blue condition.

## Discussion

From the perspective of cognitive psychologists interested in understanding human color experience, the CIE models are perfect tools for empirical stimulus specification, but they are not ideal as models of the many ways humans experience and interact with color. Our results show that CIE color differences alone are insufficient to describe color similarity when strong sources of cognitive influence on color judgment are at play. This is because CIE formalizations were not designed to predict a variety of behavioral outcomes that arise from human color appearance processing. The formal geometric properties of CIE space do not very accurately predict human judgments of, for example, color similarity, color preference or color difference in complex scenes. The results presented here suggest that a family of models that use CIE distances in conjunction with other known perceptual and cognitive factors is a promising approach to using CIE formalisms as a basis for the quantitative modeling of cognitive color relations.

### The Hierarchical Nature of Our Model

The present results confirm the suggestion (e.g., [10]) that the CIE formalization most frequently used to model observer’s judgments of color similarity and difference, is but one, albeit important, type of factor contributing to color choice behavior. To illustrate this we used features of color similarity (represented by distance in a “perceptually uniform” CIELAB model) as a predictor of choices in color triad tasks. We found that odd-one-out predictions based on CIELAB distances coincided with a good portion of the empirical data, and that above and beyond this, other plausible factors, dimensions and attributes were found to contribute. When formally included in the model, these additional factors were found to substantially improve the predictions of the triad choice behavior.

Our model can be considered a hierarchical model in the following sense. It includes the null model (the CIE distance model), which most of the time comprises the largest contribution to the choice probability. Other factors, although present, in the majority of cases do not have enough weight to modify the prediction given by the CIE distance model. In some cases, however, the CIE prediction is “weak”, which happens for example in the case when the triad is a nearly equilateral triangle. In this case, other linguistic or cognitive layers, such as category criteria or lightness-saturation baises, become the major basis for color similarity choices, and can determine the color choice behavior. These findings and this hierarchical modeling approach are related to *lexicographic semi-order* modeling of choice behavior [16]. In [16] Tversky states:

“… *When faced with complex multidimensional alternatives … it is extremely difficult to utilize properly all the available information. Instead … people employ various approximation methods that enable them to process the relevant information in making a decision. The particular approximation scheme depends on the nature of the alternatives, as well as on the ways they are presented … The lexicographic semiorder is one such approximation …* ” ([16], p. 46).

Analogous to a lexicographic semi-order interpretation of these results, we found that people initially compare CIE distances as a first approximation to their triad choice decision. When the pair-wise distances among the three items compared were different enough, then people chose the most distant item of the triple as the odd-one-out. However, if the distance-based choice becomes ambiguous, then people could engage additional criteria in making a choice. This is in the same spirit as the model of interpoint-distance color category formation described previously [28], [29].

Modeling color similarity judgments as a series of successive approximations that contribute to choice outcomes is, we believe, (i) a new and highly plausible approach to modeling the perceptual-cognitive-pragmatic aspects of stimuli that naturally play a role in color similarity judgments, and (ii) is an appropriate and useful extension of the formalisms presented by lexicographic semiorder modeling.

### CIE as a Basis for Our Modeling Approach

It should be noted that in this paper we emphasize a modeling approach that is based on the most widely used index of color difference: CIELAB ΔE. We examined alternative models based on other more recently introduced CIE color difference equations (CIE94 and CIEDE2000, see Text S1) but these alternatives either were not too different from that found with CIELAB ΔE, or they did not produce much improvement (or decline) beyond that shown with CIELAB (resembling trends seen when color difference equations are compared in applications across a range of datasets).

One way to improve the hierarchical cognitive modeling approach introduced here may be to build on one of the more complex parametric models of color appearance (i.e., CIECAM97, CIECAM02, CIECAM02-UCS) that have been developed by CIE scientists in the last decade. Although these more recent color appearance models (CAMs) strive to advance color difference estimation beyond CIELAB-based formulas, they remain under active development and are not considered final models, as they continue to evolve, and continue as topics of ongoing testing, debate, and discussion [30].

Finally, the CAMs are dramatically more complex computationally, to the point of bordering on being impractical for some applications. This is likely the reason they have not been embraced widely as tools in the empirical research literatures that study behaviors linked to cognitive and perceptual color relations. These two reasons underlie our choice to emphasize and build on the accepted, widely used, color difference index CIELAB ΔE. However, once CAM development is finalized and vetted by CIE experts, a potentially useful pursuit might be to generalize our hierarchical approach by using a CAM as the starting point for computing distances – this is entirely compatible with the aims of the present paper, but is necessarily a task for future modeling research.

### The Color Space is Non-uniform with Respect to Category and Lightness-saturation Biases

Our analyses show that different warm-cool category and lightness-saturation emphases are found across global, red and blue triad choices (Table 1). What is the reason for this difference? One possibility is that such constructs may not be uniformly appropriate, or equally relevant, across the entire color space. For example, for some regions of color space, a categorical distinction of warm-cool may be a useful or meaningful distinction to make (e.g., [31], [32]), and in other regions of the space, or for other subsets of stimuli, it may be less useful. Variation in the appropriateness of a given categorical distinction across color space can be linked to a range of factors outside the domain of the standard observer model that are likely to contribute to similarity judgments of color. Because such factors can be cognitive, cultural, linguistic, and so on, they are almost certainly beyond the scope of CIE modeling alone, and therefore not easily addressed even by further extending the latest, most advanced forms of CIE models. Our suggested analogy to lexicographic semiorder modeling within a hierarchy involving CIE modeling builds on CIE advances and allows many more factors to be accounted for and addressed.

Note that categorical considerations modeled here could originate from any number of sources. They could arise from normal biological bases [32] or less common biologically-based deficiencies (e.g., color vision dichromacy); from culturally specific factors [19], [20]; from putative environmental or ecological salience [33], [34]; from social color utility (e.g., highly valued purple ink used to dye royal cloth in ancient societies) ([35], chapter 62); or from language-based *marked/unmarked* color relations [36]. In such cases categorical considerations need not be applied uniformly across the entire color space, and may only apply in subregions of color space. The only feature required of the categorical consideration as formalized here is that it provide a connected set *‘A’/‘not A’* partition in the color space on which color similarity judgments can be evaluated.

If limited appropriateness of categorical considerations across color space is possible, then it is not surprising that the present data shows warm-cool category considerations improve the fit to the data for global and red conditions, while they explain less of the data for the case of the blue condition. Similarly, the criteria of lightness and saturation seem to play a role in triad choice data for the blue condition but not for the red condition or the global condition. Such patterns are plausible because stimulus sets for the three conditions were not sampled to be subjectively equal on all possible dimensions. Thus, cognitive and perceptual salience may be differently represented by samples comprising the three stimulus sets (and it is clear that the global stimulus set already dramatically differs in the hue dimension compared to the red or blue stimulus sets).

### Universal and Cultural Components of the Model

One advantage of CIE modeling that keeps formalizations tractable is that a standard observer approach to color modeling is used. This standard observer simplification does, however, limit the potential to address systematic variation in observer groups that can be linked to, for example, shared knowledge about color, which uniquely contributes to color similarity relations in observers with specific linguistic or cultural affiliations. In these cases where culturally relevant information is known, it would be beneficial to include such information as a factor relevant in color similarity judgments. Extending the modeling capabilities of the CIE in this way is one aim of the present hierarchical modeling approach.

This article considers data only from native-language speakers of English. But it is certainly true that category influences on color triad choices will differ for non-English speakers. For example, one would expect variation in lightness-saturation influences on color triad choices across ethnolinguistic groups [37]. Thus, what we consider *universal* in this framework are the identifiable perceptual and cognitively salient features of chosen triad stimuli (i.e., distance in a color space captured by the null-model), and what is *cultural* are the alternative approximations that additionally contribute to triad choices (i.e., pragmatic, environmental, individual and other culturally dependent factors captured by the additional layers in our model). The *universal-cultural* contrast differs here from that typically seen in the literature – that is, although the two forms of influence arise from very different sources, they nevertheless can both contribute to odd-one-out color similarity judgments.

Examples of how category influences can differ across language groups are readily available. Russian has two distinct linguistic categories (or two “basic color terms”) for colors that English denotes under the lexical category “blue” [7], [8]. Similarly, Hungarian has two basic terms for “red” colors (*piros* and *vörös*) [38] and Hungarian speakers use category glosses for “light” and “dark” (világos and s*ö*tét, respectively) to modify basic color terms much more frequently than that seen in native English color naming. This suggests that there are differences of lightness dimension salience between native-language Hungarian and English color naming [39]. Almost the opposite scenario is found in native-language Vietnamese speakers where one category term (i.e., *xanh*) is used for denoting all “greens” and “blues” and the linguistic differentiation of green appearances from blue appearances is only achieved by modifying terms that specify which *xanh* appearance is “green” (or x*anh lá cây* or “*xanh* like the leaves”) and which *xanh* is “blue” (or *xanh nu'ó'c bi
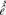
n* or “*xanh* like the ocean”) [13].

Thus, linguistic and cultural factors could set which criteria are most attended to in color similarity judgments compared across cultures. Indeed, many forms of *linguistic markedness* could influence which criteria color similarity is based on, and these would be expected to vary across ethno-linguistic groups and might even arise due to subgroups within a linguistic culture, such as color experts (e.g., professional artists and designers) compared to individuals in the same ethno-linguistic culture with little color expertise.

## Materials and Methods

Data from 52 native English speakers (32 female and 20 male) are analyzed (see Text S1 for details). Participants were randomly presented series of triad trials comprised of three precisely rendered color stimuli. In a given triad trial participants must identify which of the three items presented is most different from the remaining two. Color appearance triads were judged separately for the three color sets representing global, local red, and local blue color conditions. Each condition included 21 stimuli for a total of 70 triad judgments per condition. All experimental methods, procedures and stimuli were reported previously [11]. All further details are provided in Text S1.

## Supporting Information

Text S1 **Methods.**
(PDF)
