# Supplementary material for: A Quantitative Theory of Human Color Choices
Source: PLoS One. 2013 Feb 11;8(2):e55986. doi: 10.1371/journal.pone.0055986 (PMC3569434; doi:10.1371/journal.pone.0055986)
Supplement: Text S1 — Methods. (PDF) [file pone.0055986.s001.pdf]

Text S1

Quantitative theory of human color choices

N.L. Komarova and K.A. Jameson

1 Experimental methods

1.1 Participants

Participants were 56 subjects (34 female and 22 male) recruited either through the University of California, San Diego, Department of Psychology human subjects pool, or by posted solicitations. Participants received either cash payment or course extra-credit. All subjects were native speakers of English. The study was approved by University of California, San Diego Human Research Protections Program (HRPP) Institutional Review Board (IRB). Written informed consent was obtained by all participants in accordance with UCSD Human Research Protection Program (HRPP) protocol. The IRB ethics committees approved all aspects of the recruitment and consent procedures used. Copies of written informed consent forms with identifying subject information redacted are available. Four subjects (2 male, 2 female) were omitted from data analyses due to procedural errors during data acquisition. The remaining 52 subjects (32 female, 20 male) completed all experimental tasks. Subsequent color vision screening identified four of the male participants as having some form of color vision anomaly, see below.

1.2 Apparatus and procedure

Stimuli were generated by a PowerPC Macintosh 7200 and displayed on a Hitachi RasterOps MC 7515, 21" CRT monitor with a 19" viewablediagonal and EBU monitor phosphors. CIELUV 1976 (u*,v*) values for phosphors were Red (.4507, .5230); Green (.1206, .5610); Blue (.1754, .1580). The screen resolution was 1024x786 at 70 Hz (24 bit color). The experimental display was checked regularly for accurate color rendering using a calibration measurement system dedicated to colorimetry functions. Blackout material covered surrounding CRT casing leaving only viewable screen area. Stimuli were manipulated on the screen via a trackball mouse and responses were recorded using a PsyScope button box (Carnegie Mellon University, Pittsburgh, PA). Experimental procedures were original routines implemented using PsyScope 1.2.5 PPC software (Cohen, MacWhinney, Flatt, and Provost, 1993).

Participants were dark-adapted for at least 10 min. in a dimly lit room diffusely illuminated at 2 Lux by a halogen lamp. The instructions were visibly displayed and the experimenter read all task instructions to subjects. Practice trials preceded data collection. In the experiment participants were randomly presented series of triad trials comprised of three precisely rendered color stimuli. In a given triad trial participants must identify which of the three items

presented is most different from the remaining two. Color appearance triads were judged separately for the three conditions tested (i.e., global, local red and local blue). Each condition included 21 stimuli in a *Balanced Incomplete Block Design* (BIBD, lambda = 1) for a total of 70 triad judgments per condition.

A Balanced Incomplete Block Design (BIBD or BIB) is an established way of reliably assessing a subset all possible triples among a number of items [20]. BIBDs have been used extensively in the study of semantics in anthropology, clinical psychology and psychophysics as a technique for deriving information about structural relations among many items [4]. Such designs are important tools in investigations of cognition, and can be traced back to mathematical psychologist C. H. Coombs [5]. Triad BIB designs are proven as alternatives to presenting participants a full complement of triads. Here a full complement of triadic comparisons for $N = 21$ items for a single conditions would be 1330 triads (given by $N(N-1)(N-2)/6$, where each pair is presented with every other item in the stimulus set). Requiring 1330 judgments for each of the three conditions we tested would be empirically impractical, and would be expected to have undesirable impacts on participant performance. The $\Lambda = 1$ BIBD we employ uses 70 triads to assess 21 stimulus items where each pair of items is presented in a triad one time. By design, this is a sparser sampling than a complete design ($\Lambda = N - 2$) or a $\Lambda = 2$ or $\Lambda = 3$ design (where each pair of items occurs twice or three times). We however can be confident of the robustness of the data produced, because this methodology has been employed elsewhere to recover relational structure for other stimulus domains and has been tested extensively [**?**]. More importantly, in the present data for all three conditions tested we observed a high degree of shared agreement (i.e., well-correlated observer response patterns) in the triad color judgments of participants, as measured by high average consensus levels (near 0.7), and, as described in detail elsewhere, the data in all 3 conditions meets all Consensus Theory criteria [16] for a robustly shared knowledge domain [17]. Thus, while potential for increasing noise and random errors exists in essentially any incomplete empirical design, we are confident the BIBD we use more than adequately captures the desired structure from the color similarity phenomena we model in this paper.

1.3 Stimuli

Stimulus selection was guided by aims of (1) studying color similarity judgments using comparatively larger sets of color stimuli than that previously used in similar cognitive research [13]; (2) investigating color categories identified as "Basic Colors " [1]; (3) maximizing the potential observation of color naming variation across different observer groups [10]. Of these 3 aims only the first is emphasized in the present paper (see Sayim et al. [17] for additional results). Three stimulus sets from 3 regions of color space were selected. Each set consisted of 21 color samples (specifically, 21 stimuli gave an acceptable number of triad trials in a balanced block design) with associated color names (the color name results are reported in [17]). Stimuli were chosen following a principled procedure that produced a representative sampling of color space for the three color conditions

investigated. Three conditions consisted of 21 "global" colors, 21 "local red" colors, and 21 "local blue" colors. Global color stimuli included eight OSA *centroids* identified earlier as corresponding to eight salient color terms [3]. The remaining 13 global color stimuli corresponded to OSA tiles named with the highest frequency in a study of unrestricted naming in English of all 424 OSA tiles [6]. Local stimuli were selected to permit comparing similarity structure of within-category conditions with that for global (across-category) stimuli. By studying local red and blue color stimuli, we aimed to elicit potential differences between the global and local conditions, following the rationale that color similarity judgments should differ for local and global stimulus sets [9]. Red and blue categories were also chosen to explore a potential difference between observers with varying color vision capabilities (as aspect of the research presented elsewhere [17]). A general selection heuristic was used to select red and blue stimuli: the monolexemic naming data of Boynton and Olson[3] was used to identify 21 OSA tiles, from each category (red and blue), that were reliably named "red" and "blue" by a majority of their subjects. Minor deviations from this strategy were needed and are described elsewhere [17]. Figure 2 of the main text shows an approximation of the 21 colors sampled for each condition. After triad data collection subjects answered demographic questions and were screened for color vision abnormalities. All details of the experimental procedure are found in [17]. One triad example from each stimulus condition is shown in figure 1.

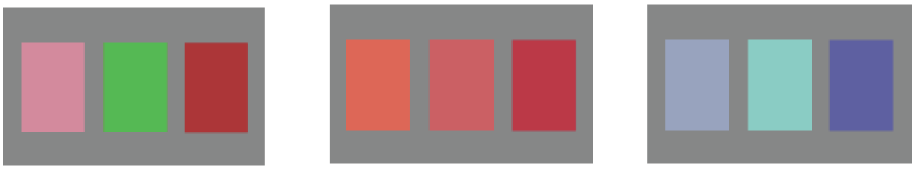

Figure 1: Examples of triad stimuli used for the global condition (left), red condition (middle) and blue condition (right). Colors shown are only approximations of that presented during the experiment on the calibrated color CRT display.

2 Color difference based on distance measurements

2.1 Different distance models

To evaluate the predictions of different color distance models, we use the following method. For each condition (global, red, and blue), for each triad we calculate the three color distances among the three stimuli. The stimulus opposite the shortest of the distances corresponds to the predicted odd-one-out choice [2, 15]. For each triad, such theoretically predicted choice is compared to

the majority choice of the responders. If the majority of the group picks a stimulus different from the predicted one, we call this a "mismatch". The number of mismatches (out of the 70 triads for each condition) characterizes roughly how well a color distance metric alone can predict the observers' behavior.

Three distance models have been used and indices of color difference:

1. **CIELAB.** The first distance model [7] is the Euclidean distance measured by using the three CIELAB coordinates for the two stimuli, L_1, a_1, b_1 and L_2, a_2, b_2:

$$\Delta E = \sqrt{(L_2 - L_1)^2 + (a_2 - a_1)^2 + (b_2 - b_1)^2}.$$

2. **CIE94.** The second model we used is the CIE94 delta-E distance formula [8]:

$$\Delta E = \sqrt{(L_2 - L_1)^2 + \frac{\Delta C^2}{(1 + 0.045C)^2} + \frac{\Delta H^2}{(1 + 0.015C)^2}},$$

where we defined

$$\begin{aligned} C &= 1/2 \left(\sqrt{a_1^2 + b_1^2} + \sqrt{a_2^2 + b_2^2} \right), \\ \Delta C &= \sqrt{a_2^2 + b_2^2} - \sqrt{a_1^2 + b_1^2}, \quad \Delta H = \sqrt{2(C^2 - a_1 a_2 - b_1 b_2)}. \end{aligned}$$

3. **CIEDE2000.** Finally, we used the distance defined by the CIEDE2000 formula [11] where we employed the implementation algorithm given in [18]. The appropriate formulas are rather lengthy and we refer to the above sources for the mathematical expressions. CIEDE2000, was recommended by the CIE in 2001. It includes five corrections to CIELAB: a lightness weighting function, a chroma weighting function, a hue weighting function, an interactive term between chroma and hue differences for improving the performance for blue colors, and a factor for re-scaling the CIELAB a* scale for improving the performance for neutral colors[12]. Through such adjustments some configural visual processing effects, like "crispening", are addressed. While CIEDE2000 is the newest color difference formula recommended by the *Commission International de l'Eclairage*, it is not a comprehensive color appearance model, similar to CIELAB and CIE94, but it is by far the most parsimonious and it provides color difference measures that have been empirically shown to be statistically similar (within suggested levels of STRESS $\leq$ 5.0 [**?**]) to the most recently developed CIECAM02 family of color appearance models (see [19], p. 324 Table XI). We use CIEDE2000 here due to its straightforward, nonparametric computational form, and because we considered it a more practical alternative (i.e., more likely to be used by researchers in color cognition and industry) to the more advanced CAM models. The latter models strive to incorporate important theoretical parameters, but they still remain computationally onerous and untested with respect to their appropriateness as metrics for cognitive science research on color behaviors or color similarity.

Table 1 shows the results of the application of the above three methods to evaluate the number of mismatches in each condition. In particular, the following patterns have been observed:

- For each condition, all three methods give similar numbers of mismatches.
- For each method, the number of mismatches in the blue condition is the largest.
- CIEDE2000 formula give the smallest number of mismatches in all three conditions.
- The total number of mismatches is the smallest for CIEDE2000 and the largest for CIE94.
- The sets of mismatches produced by the different methods overlap significantly. The number of mismatches common between CIEDE2000 and CIELAB method is 7/12, 11/13, and 16/17 for the three conditions; the number of mismatches common between CIEDE2000 and CIE94 method is 12/12, 12/13, and 14/17 for the three conditions (these data do not appear in the table).

	CIELAB	CIE94	CIEDE2000
Global	13	17	12
Red	15	17	13
Blue	24	20	17
Total	52	54	42

Table 1: The number of mismatches resulting from the three color distance models, for each of the three experimental conditions.

2.2 The null-model of color choice

In order to evaluate the use of CIE color difference metrics, or color space distance, as a general measure of color similarity, we performed the following analyses. All 210 of the triad stimuli from the global, local red and blue conditions differ with respect to the metric distances their triangles span in a color space. Some of the global triads are comprised of one or more color stimuli separated by large distances across color space, whereas some local triads are comprised of stimuli that are, by comparison, relatively near each other in the color space. When some stimuli in a triad are very distant in color space this can make the odd-one-out choice easier, or more certain, if the choice is based purely on distance. Thus for the 210 triad stimuli used in the experiments we constructed a *certainty index* for classifying gradient levels of triad certainty based on CIE

distances described above. For each triad, we calculate its certainty index, given by

$$C_{cert} = \frac{2(o_2 - o_1)}{o_2 + o_1},$$

where $\{o_1, o_2, o_3\}$ is the ordered set of the triad's side lengths, in the increasing order. The certainty index conveys the relative difference between the two shortest triad side lengths. A small certainty index reflects very ambiguous (uncertain) triads, and a large certainty index reflects non-ambiguous triads, or triads with greater choice certainty for the most distant stimulus in that triad. Based on these calculated certainty indices, all 210 triad stimuli are grouped into 10 classes; we call these groups "certainty classes".

We postulate the following null-model of behavior. Given a triad $\{o_1, o_2, o_3\}$, an observer will pick stimulus i with probability

$$p_i = \frac{1}{s o_i^\alpha}, \quad s = \sum_{k=1}^{3} \frac{1}{o_k^\alpha}, \quad i = 1, 2, 3. \tag{1}$$

In other words, the probability to choose a given stimulus is inversely proportional to (the power α of) the length of the opposite side of the triad, measured in terms of the CIE distance. This model reflects some intuitive geometric properties of triads. For example, a triad with two equal sides which are shorter than the third side will be very ambiguous, and correspond to $p_1 = p_2 > p_3$, that is, two stimuli will have an equal probability to be picked. On the other hand, a non-ambiguous triad with one very short side and two long sides will have a very large probability for the correct stimulus choice.

The parameter α measures the amount of intra-individual variation. Large values of α mean that the same correct stimulus will be picked almost certainly. Small values of alpha $\alpha \approx 1$ correspond to the situation where the "second" and "third best" choices will also have a measurable probability to be picked, which can be the result of sloppiness and inconsistency of observers. The trivial case $\alpha = 0$ corresponds to $p_i = 1/3$, that is, all triads have an equal probability to be picked. It will not be considered here; instead we will concentrate on the range $\alpha \in [1, 5]$. In all the models (except for $\alpha = 0$), the CIE-predicted stimulus choice has the largest chance of being chosen. Figure 2 illustrates the role of parameter α in the model. For each triad, we calculated $1 - p_1$, which is the probability for an observer modeled by equation (1) to pick the "wrong" stimulus. These values are presented for different values of α. We plot the quantity $1 - p_1$ as a function of the certainty index, C_{cert}, for all 210 triads (the plot in 2(b) shows the same information as 2(a), but on a log-scale). We can see that, not surprisingly, the triads with a larger certainty index are characterized by a smaller probability of error. Further, the probability of error decreases with α. In other words, large values of α in model (1) mean that the observers are very self-consistent. Smaller values mean an increased amount of "sloppiness" and inconsistency.

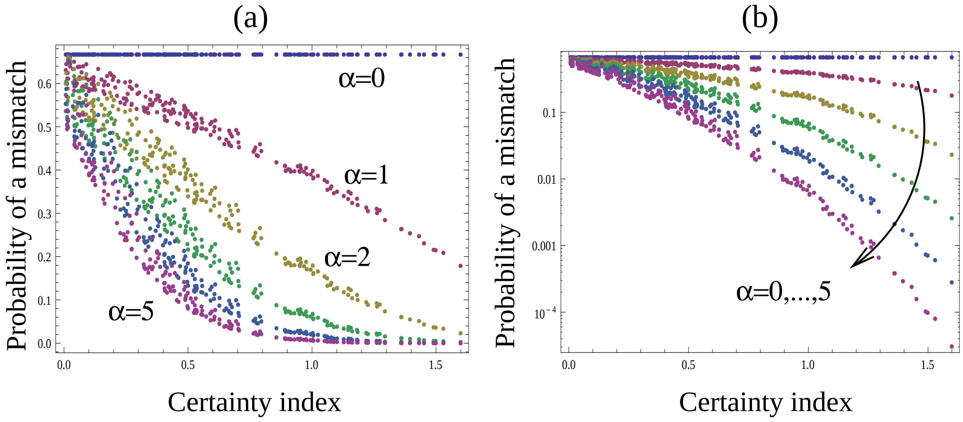

Figure 2: The role of parameter α as a measure of intra-individual differences of subjects. (a) The probability of a mismatch, $1-p_1$, for all the 210 triads, as a function of the certainty index, for $\alpha=0,\ldots,5$. (b) Same as (a), but on a log-scale, to show detail.

3 Statistics of triad responses

To understand how choice data varies across the 52 observers, we compare responses from different subsets of our sample. On one hand, we consider the aggregate data from the total group of observers (all 52 participants). And, on the other hand, we consider the 52 individual observers (which for the sake of comparison are analyzed as if they were 52 separate "groups"). We also considered intermediate-size groups, for example, all the groups of two observers, etc. For each subset of the population, we determine the majority vote for each triad, and compared that with the CIE prediction for the same triad. Then we count how many mismatches are in each certainty class. In figure 3 we present the number of mismatches found for the group of 52 observers (blue circles, marked as "Real observers, set of 52"), and the average of the 52 "groups" of one observer (orange dots, marked as "Real observers, sets of 1"), as a function of the certainty index. We observe the following patterns:

- The percentage of mismatches decreases as a function of the certainty index.
- The percentage of mismatches is higher for smaller observer subsets than it is for larger observer subsets.

To model the individual and group responses, we will discuss several sources of variation that could occur. The different types of variation can be characterized as follows:

1. **Sampling, or intra-individual variation**. These variations arise as random chance events, and will be more prevalent among the ambigu-

ous triads. These variations are reflected in observer test-retest inconsistencies, can be ascribed to individual behavior, such as sloppiness in completing the triad test.

2. **Inter-individual variation**. These variations reflect (a) inhomogeneities of the observers (the presence of a small number of dichromat or anomalous trichromat observers), or (b) individual cognitive factors such as personal color salience or preference.

3. **Systematic variation**. These variations can arise (a) from inconsistencies of the CIE description of the perceptual space, or (b) from some systematic cognitive factors such as conventions. These variations reflect deviations from the CIE prediction which are common among a large portion of the population.

The three classes of variation listed above have very distinct features, and in the following sections we discuss their mathematical properties. We show that variations of type (1) and (2) alone cannot explain the observed variation, and one must assume the existence of some systematic source of variation, (3).

3.1 Sampling and intra-individual variation

To consider this type of variation in isolation, we assume that all individuals have exactly the same perceptual ordering of stimuli across color space, and this space is correctly described by a CIE model of color distance. We will not include any personal preference or individual differences in the description at this stage. The observers described here can be called "idealized observers", because they are identical to each other, and because their perceptual space is described accurately by a CIE color distance formula together with our model (1).

Given this behavioral model, it is obvious that even with a population of identical observers, there is a possibility of mismatches, which is larger for smaller values of α, see previous section. Even though for each individual and for each triad, there is a stimulus that will most likely be picked, there is still a probability of picking a suboptimal stimulus. The amount of this effect is incorporated in the parameter α.

For a given value of α in formula (1), we can calculate the probability that the majority of the population will pick the CIE-predicted stimulus. Let us suppose that there are k people in the subset, and defined the quantity $k^{(1/3)}$ to be the smallest integer such that $k^{(1/3)} \geq k/3$. Then the probability for the majority to pick the CIE-predicted stimulus is given by

$$P_1 = \sum_{i_1=k^{(1/3)}}^{k} \sum_{i_2=k-2i_1}^{i_1} \frac{k!}{i_1!i_2!(k-i_1-i_2)!} p_1^{i_1} p_2^{i_2} p_3^{k-i_1-i_2}. \qquad (2)$$

In derivation of this expression we used a non-strict definition of majority: if the number of people choosing stimulus i is given by k_i with $\sum_{i=1}^{3} k_i = k$, then

k_i is a majority if $k_j \leq k_i$ with $j \neq i$.[1] In the above formula we assume formally that the factorial of negative integers is infinite. Under this assumption we see that with $k = 1$, we simply have $P_1 = p_1$.

Let us suppose that a given certainty class contains m triads. Our theory predicts how many mismatches we can expect in a population of k idealized observers, and the variance of this amount:

$$E(mis) = (1 - P_1)m, \quad Var(mis) = (1 - P_1)P_1 m, \tag{3}$$

according to the binary distribution.

In Figure 3 we illustrate the behavior of the idealized observers. The horizontal axis corresponds to the certainty index, and the vertical axis is the number of mismatches for each certainty class. The total number of triads in each class corresponds to our 3 experimental conditions with a total of 210 triads. Figure 3's two curves with error bars are the predictions of our theory with the idealized observers. The green curve corresponds to $k = 52$ observers and the red curve to $k = 1$ observers. We plot both the expected number of mismatches and the standard deviation. We can see that the case with $k = 52$ observers corresponds to a much smaller expected number of mismatches. It is easy to show that as $k \to \infty$, the number of mismatches of the population majority will also tend to zero. This is an inherent property of sampling variation of this kind.

In our model, α is an unknown parameter, which measures the averaged observer consistency, and which we can choose to make our model match the data as close as possible. In figure 3 we chose the parameter $\alpha = 2$ such that the curve corresponding to the individual real observers lies close to the theoretical curve of sampling variation. As we can see however, the curve corresponding to the group of 52 real observers does not match the sampling variation prediction. Taking different values of α does not improve the situation. The problem is that in the presence of sampling and intra-individual variation only, the population of 52 observers performs a lot better (that is, has much fewer mismatches) than populations of 1 observer. That is, the amount of sampling variation decays to zero very fast as the group size increases. This is not the case with the real observers, whose $k = 1$ and $k = 52$ curves are relatively close to each other. From this we can conclude that sampling/intra-individual variation alone do not describe the data completely.

Before we move onto the next type of variation, we point out that in order to check the validity of formulas (2-3), we have created artificial populations of idealized observers by picking their triad responses in accordance with probabilities p_i. The population counts for these observers (in groups of 52 and in groups of 1, averaged over 20 runs) are also presented in figure 3. They are lines marked as "Idealized observers, sets of 52" and "Idealized observers, sets of 1". As expected, both of these lines are in close proximity with the theoretically predicted sampling variation lines.

[1] A strict definition of majority would require a strict inequality.

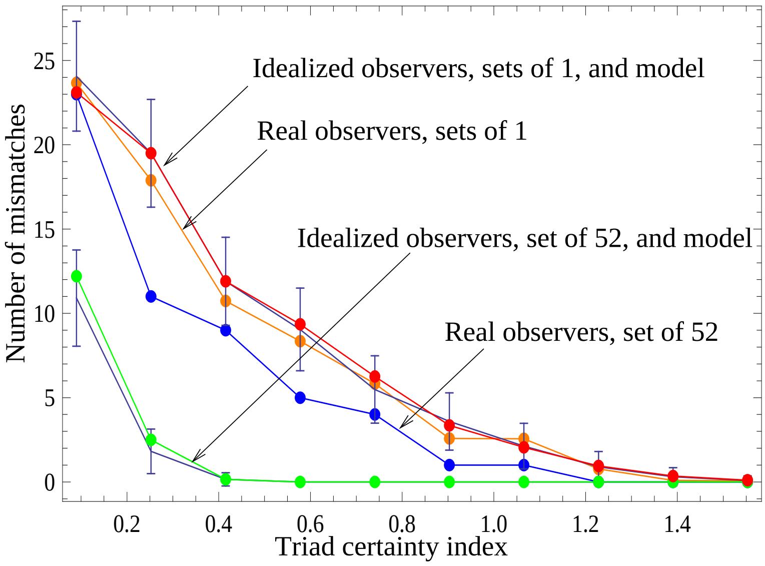

Figure 3: Sampling/intra-individual variation. The number of mismatches is presented for different triad certainty classes. Shown are the two theoretical curves for sampling/intra-individual variation, with error bars; two curves for the real observers corresponding to $k = 52$ and the average of all $k = 1$; and two curves for the average results of simulated idealized observers, with $k = 52$ and $k = 1$. The parameter $\alpha = 2$.

3.2 Inter-individual variation

An example of inter-individual variation is the presence of individual differences of perceptual space across the observers. Such differences are unique to each observer (or to small subsets of observers) and can be caused by factors such as dichromacy or other color vision features when such conditions are only present in a small fraction of individuals (otherwise, the presence of the same type of dichromacy in all individuals would have to be characterized as a systematic variation, see below). Another reason for individual variation could be individual behavioral differences, such as certain color preferences, or idiosyncratic color salience. We characterize this type of variation by creating a different perceptual space for each individual.

To model this, we assume that the perceptual space of each individual is somewhat different. The perceptual space for each observer is obtained by introducing a distortion to the CIE space. To create possible perceptual spaces, we take the coordinates of the 63 stimuli and introduce a perturbation to them, in the form $(L, a*, b*) \to (L + A(-1/2 + \xi), a* + A(-1/2 + \eta), b* + A(-1/2 + \zeta))$, where A is the amplitude of the perturbation, and ξ, η and ζ are some random numbers uniformly distributed in $[0, 1]$. We used different random numbers when perturbing the different color stimuli. Also, these numbers are different

for different individuals. In this model, the parameter A controls the maximum amount of distortion between the CIEL*a*b* coordinates and the actual perceptual coordinates used by the individuals.

Artificial observers with varying perceptual spaces are created in this way, and their data analyzed as described above. It turns out that, similar to the intra-individual variation, this type of variation does not improve our description of the observed data. Figure 4 presents two attempts to match the data with simulations of nonhomogeneous observers. In figure 4(a) we took $\alpha = 2$ and $A = 40$, to match the curve corresponding to $k = 52$. However, the simulated $k = 1$ curve overestimates the number of mismatches. In Figure 4(b), the parameters $\alpha = 4$ and $A = 20$ assure that the $k = 1$ curve is matched well; however, the number of mismatches is underestimated in the $k = 52$ case. In general, increasing A and decreasing α corresponds to an increase in the number of predicted mismatches. Manipulating these parameters over a wide range of values does not result in a satisfactory fit with the real observer data.

This analysis shows that inter-individual variation is insufficient to explain the data. This is consistent with the fact that although four out of 52 observers in the sample were identified as having some form of color vision anomaly, their presence did not significantly affect the number of mismatches between the model and the observed data. In our framework, those four individuals represent individual variation which cancels out when studying group responses.

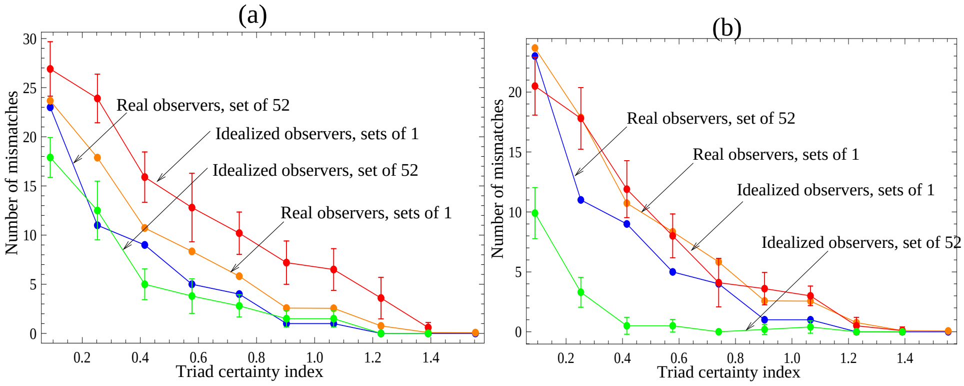

Figure 4: Inter-individual variation. Same as in Figure 5, but with nonhomogeneous idealized observers. The parameters are (a) $\alpha = 2$, $A = 40$; (b) $\alpha = 4$, $A = 20$.

The color similarity literature examines both intra-individual and inter-individual variation in triad judgment performance, as these sources of variation will diminish the goodness of fit of any color models proposed. For example, using a color triad task with 20 normal trichromat subjects, Bimler and colleagues quantified the stability of subjects' test-and-retest triad performance using multidimensional scaling analyses of individuals' color similarity space [2]. In order to to this, they defined *Dmn* an index of disconcordance be-

tween test (m) and retest (n) datasets for each individual (simply, Dmn is the straight-line distance between points, as described p. 74 of [2]). They found the average within-subject index was $Dmn = 0.26$, which is an indication of the average intra-individual variation in triad similarity scalings across repeated observations. By comparison, the average value of Dmn inter-individual variation (between subject variation) overall was $Dmn = 0.33$. They report this intra-individual variation to be significantly smaller ($p < .05$) than the inter-individual variation in color triad similarity structures ([2], p. 74).

The important point for the present paper is that while intra- and inter-individual differences in color similarity structures produced by triad performance are significantly different, they have similar orders of magnitude (see also [13]), and the intra-individual differences are smaller (albeit only slightly) than the inter-individual differences. Our experiments did not assess individual repeated observations, so we cannot directly quantify the impact of intra-individual variation on the degree to which our model fits the group data. However, as mentioned earlier in Section 1.2, in all three conditions we investigated, a high degree of shared agreement was seen across participants in triad color judgments (using Consensus Theory analyses)[16]. This provides confidence that the datasets modeled here have, as shown by an independent analysis[17], a high degree of consistency both within and across participants.

In our model, we propose a quantitative way to model the different sources of variation. We use the parameter α which is an alternative way to measure the consistency of observer responses. This measure is in a sense more universal because it allows to compare the consistency of responses across different triads, which may differ in their certainty index. To model inter-individual variations, we vary the underlying individual models of color distances of individuals. We find that inter-individual variations, like intra-individual variations, are not enough to explain the observed responses.

3.3 Systematic variation

An example of systematic variation could arise from inconsistencies in the CIEL*a*b*, CIE-delta E, or CIEDE2000 descriptions of perceptual space, or cognitive behavior patterns common to all the observers that are not included in the CIE models, such as common conventions. To model this kind of variation, we again assume that all the agents have identical perceptual space (a homogeneous group of observers), and that it is not necessarily well described by the CIE formalisms.

To model this situation we assume that the perceptual space (shared by all the observers) is different from the CIE space that we use to identify the CIE-predicted triads. This is done exactly as described in section **??**, except we create only one perceptual space, different from CIE and common for all individuals. To clarify, the difference between the CIEL*a*b* space and the perceptual space could be due to the inconsistencies inherent in the CIE model, or to cognitive factors common to all the observers.

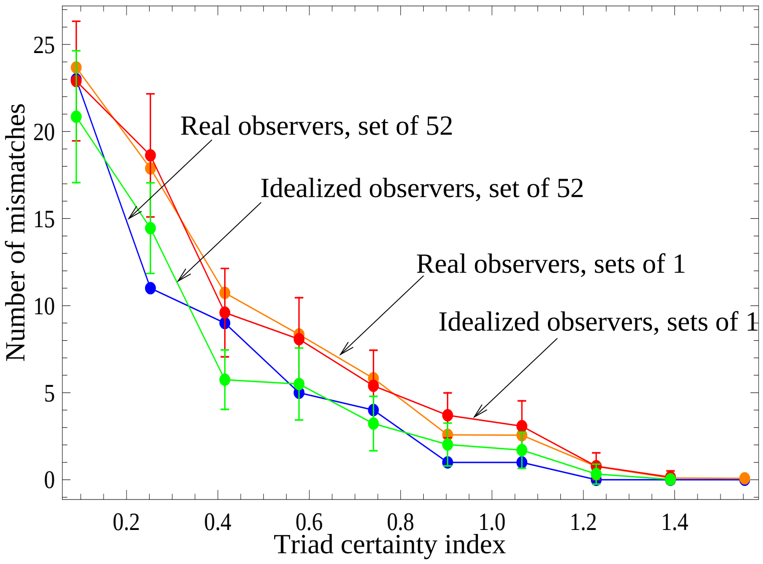

Figure 5: Systematic variation. The number of mismatches is presented for different triad certainty classes. Shown are the two curves for the real observers corresponding to $k = 52$ and the average of all $k = 1$; and two curves for the average results of simulated idealized observers with systematic errors, with $k = 52$ and $k = 1$. The latter two curves are equipped with error bars for 40 independent simulations. The parameters are $\alpha = 4$ and $A = 20$.

In order to implement this model of systematic variation, we created idealized observers who sampled their triads according to formula (1), with the triad side lengths calculated as described before, but now based on a modified perceptual space. Figure 5 presents simulation results for such observers, averaged over 40 independent simulations, with $k = 52$ and $k = 1$. As we can see, the curves corresponding to the real observers fit almost everywhere inside the error bars of the curves for the simulated observers. The parameter α was chosen to be $\alpha = 4$ for these simulations. This parameter value yields the closest fit between the simulated and real observers. We note that this parameter value is characteristic to the particular distortion of the perceptual space simulated here.

We conclude that the presence of some systematic variation is consistent with the observed response and can describe the observed variation. Compared to the systematic variation we studied, influences attributable to non-systematic intra- and inter-individual variations have decreased impact on the fit of our models to data when overall group averages were considered, and can be thought as similar to the addition of random noise to the data. A consequence of this finding is that modeling average data which does not have any systematic variation underestimates the number of mismatches for larger group sizes, and the

underestimation grows as group size increases.

3.4 Further analysis of observer responses

So far we have been concentrating on the analysis of mismatches. While this is a useful way to compare the predicted and actual experimental result, the number of mismatches does not include all the information about the responses. Another quantity associated with the observer responses which does not only include the majority vote, is the consistency index. It is constructed similarly to the certainty index of the triads, but instead of using CIE distance measurements it uses the observer responses. Let us suppose that the numbers $\{k_1, k_2, k_3\}$ is the ordered set of the numbers of people who picked the three different stimuli in a given triad, where $k_1 \geq k_2 \geq k_3$. We have $\sum_{i=1}^{3} k_i = k$, and k_1 is the number of people who picked the most "popular" stimulus. We define the consistency index, C_{cons}, as follows,

$$C_{cons} = \frac{2(k_1 - k_2)}{k_1 + k_2}.$$

If C_{cons} is small then the top choice stimulus was a "close call". A large consistency index indicates that the choice was made by a vast majority of the people.

We can represent each triad item as a point in a 2D space (C_{cert}, C_{cons}). In Figure 6 we present all the 210 triads as points in this space; the triads corresponding to the mismatches (corresponding to the $k = 52$ population) are denoted by larger points. Figure (a) corresponds to the real observer data, and the other three figures show our simulated data, where (b) contains sampling/intra-individual variation only, (c) the systematic variation and (d) the inter-individual variation.

We can see that the point distribution in figure (c) (systematic variation) matches the real data (a) much better than the other two figures. In particular, with sampling/intra-individual variation only (Figure 6(c)), we observe that the certainty and consistency indices are much better correlated than in the real data. This is of course to be expected, because "incorrect" responses are more likely for ambiguous triads, and the only source of errors is the triads' ambiguity. The inclusion of inter-individual variation still preserves this high degree of correlation. This is because inter-individual variations largely cancel out when we are considering responses of a relatively large number of observers. Therefore, for such populations, the major source of variation is again the sampling variation.

We conclude that a necessary component in model that makes it consistent with the observations is the presence of systematic variation. Inter- and intra-individual variation are secondary factors, which alone are insufficient to explain the data.

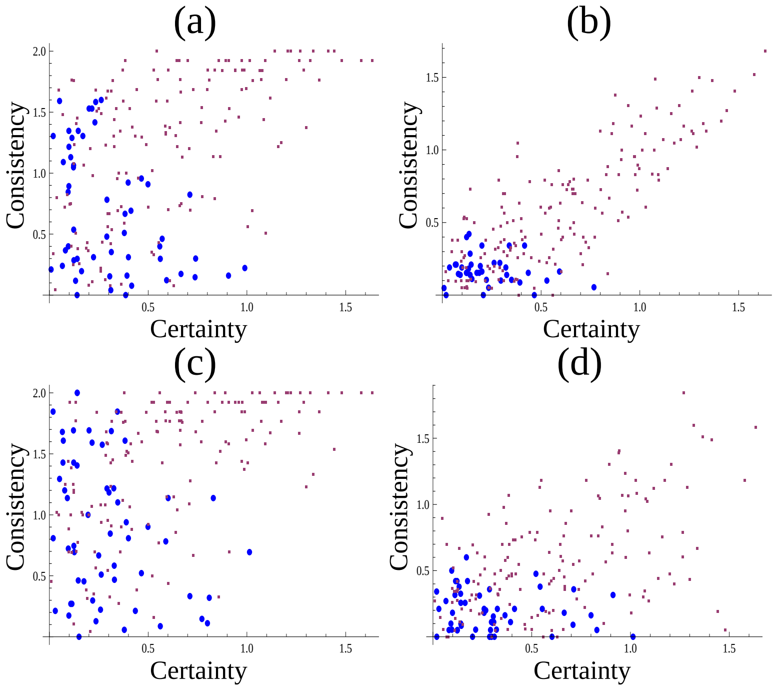

Figure 6: Triads in the certainty-consistency space, (C_{cert}, C_{cons}). (a) The real observer data; (b) sampling/intra-individual variation only, with $\alpha = 1$; (c) systematic variation, parameters as in figure 5; (d) inter-individual variation, parameters as in figure 4(b). The larger points correspond to the mismatched triads.

4 The expanded model: categorical and lightness-saturation biases

4.1 Procedure

In order to incorporate category biases, we performed the following procedure by using Mathematica software. First, the stimuli in each of the experimental conditions (red, blue and global) were projected onto the hue-saturation plane (the $a*b*$ plane), and their $a*b*$ values converted into the polar coordinates, such that each stimulus is characterized by (r_i, ϕ_i), a radial and an angular coordinate for $1 \leq i \leq 21$, the 21 stimuli in each condition. Then, the procedure is slightly different for the local conditions and the global condition.

In each of the local conditions, two angles φ_{min} and φ_{max} were identified, such that $\varphi_{min} = \min_{1\leq i\leq 21} \phi_i$ and $\varphi_{max} = \max_{1\leq i\leq 21} \phi_i$. A step-size $\Delta\varphi = (\varphi_{max} - \varphi_{min})/N$ was defined for some large integer value N (the larger the number N, the more refined the analysis). Then angle φ was varied between φ_{min} and φ_{max} with step $\Delta\varphi$. For each value of φ, a radial line corresponding to

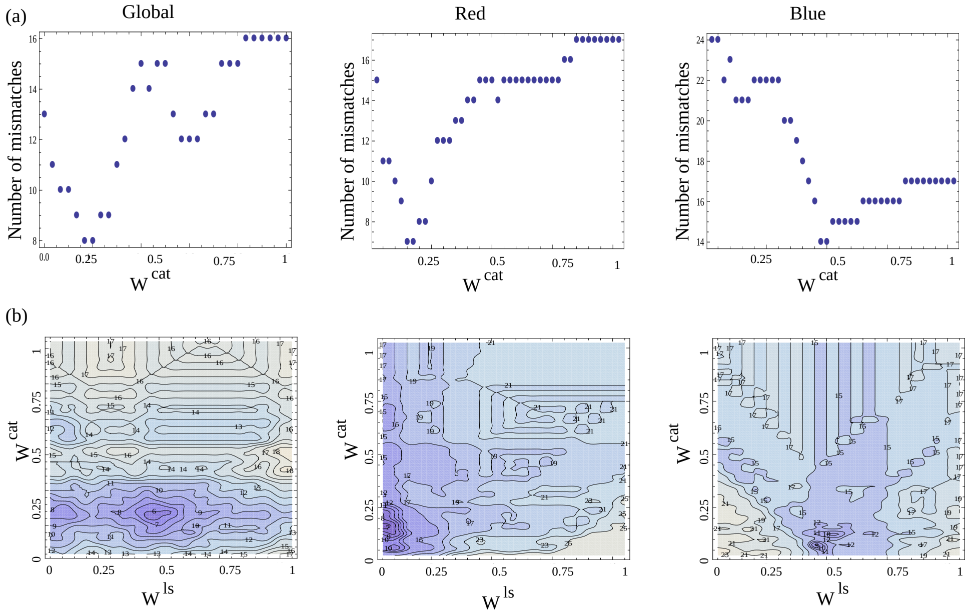

Figure 7: Color distance model CIELAB: the optimization problem. The number of mismatches as a function of parameters W^{cat} and W^{ls}. The top row (a) presents model (2) where only categorical bias is included ($W^{ls} = 0$). The bottom row (b) shows contourplots of the number of mismatches as a function of the parameters W^{cat} and W^{ls} for the full model (3). Results are presented for the global condition (left), the red condition (middle) and the blue condition (right). The parameter $\alpha = 2$.

the angle φ split the stimuli into two groups, which were treated as "categories". For each fixed value of φ, we then performed the optimization of the weight parameter, W^{cat}. To do that, we varied W^{cat} in formula (2) of the main text between 0 and 1 with a small increment. For each given value of W^{cat} we calculated the number of mismatches yielded by the model, resulting in a figure similar to figures 7(a). The value of W^{cat} corresponding to the smallest number of mismatches was noted. Then the same procedure was repeated for the next value of the angle φ. In the end, the value of φ which resulted in the smallest possible number of mismatches was identified. The corresponding categories are presented in figure 2 of the main text, and the number of mismatches for this value of φ are shown in figure 7(a) as a function of the parameter W^{cat}. The smallest number of mismatches obtained appears in Table 1 of the main text.

A similar algorithm was used for the global condition. The only difference was that instead of one angle φ splitting the stimuli into two "categories", we used three values, φ_1, φ_2, and φ_3, which split the 360° field of all stimuli in the global condition into three categories. The values $\varphi_1 < \varphi_2 < \varphi_3$ were varied independently. The configuration presented in figure 2 of the main text

corresponds to the smallest number of mismatches, see also Table 1.

This procedure was repeated for several values of the parameter α. It turned out that the exact value of α did not make a significant difference.

Next, we added the lightness-saturation biases, formula (3) of the main text. Now, instead of just one weight parameter, W^{cat}, we worked with two parameters, W^{cat} and W^{ls}, which were varied independently in the two-dimensional space, see figure 7(b). The number of mismatches is now minimized over a two-dimensional domain, yielding the best fit for both W^{cat} and W^{ls}.

4.2 Results for CIEDE2000

In the main text of the paper we present the results of these calculations in the context of CIELAB color distance formula, primarily because CIELAB is most widely employed in practice (by color cognition and perception scientists and in industry) and is reputed to be an approximation of color appearance space that is thought to work fairly well empirically. We have also performed the same calculations for the CIEDE2000 color difference formulae. formula which has been shown The optimization process for the weights W^{cat} and W^{ls} for the three experimental conditions is illustrated in figure 8. In figure 9 we show the optimal categorization solutions for the CIEDE2000 color difference formulae.

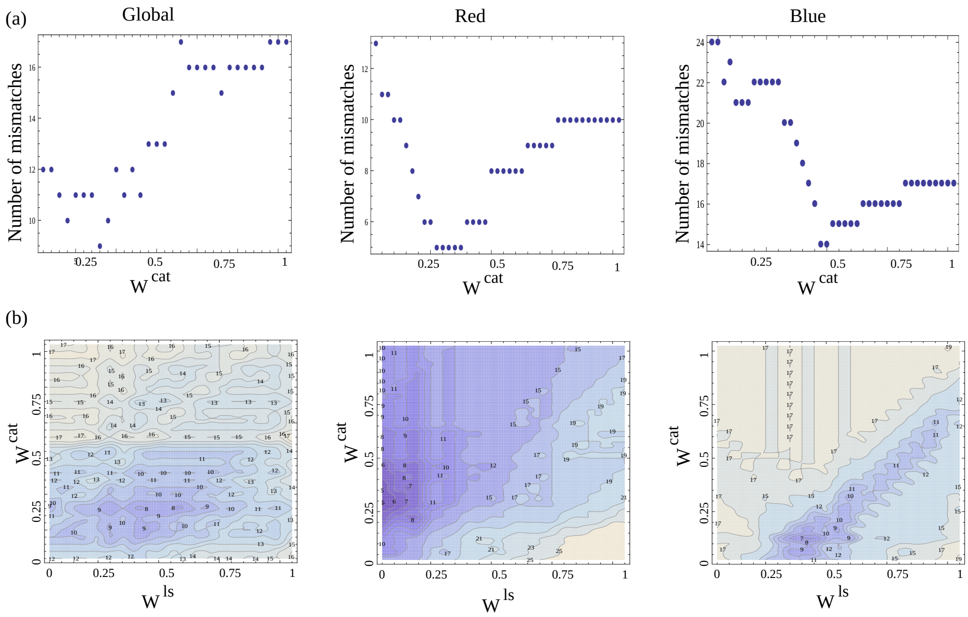

Figure 8: The same as in figure 7, except the calculations are performed using the CIEDE2000 color difference formulae.

In table 2 we present the results of using extended models in the context of the CIEDE2000 color difference forumulae, in the three experimental conditions.

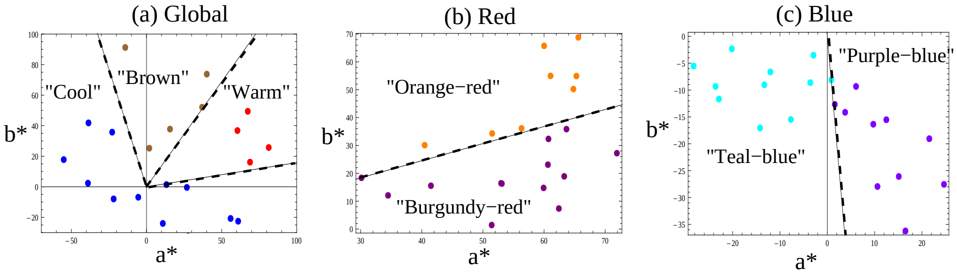

Figure 9: Optimal category choices for CIEDE2000 color difference formulae, in the three experimental conditions, global (a), red (b) and blue (c). The 21 stimuli in each condition are plotted on the a*b* plane. The categories are marked by different colors (which do not correspond to any real colors used), and separated by radial dashed lines.

	Global	Red	Blue
Null	12	13	17
Null+category bias	9	5	16
Null + Category +LS bias	8	5	7
Null + LS bias	12	13	9

Table 2: The number of mismatches using CIEDE2000 color difference, for the three experimental conditions, for the null model and the extended models.

We observe the following trends:

General. Including additional biases, such as categorization bias and lightness-saturation bias, result in a decrease of the number of mismatches in all three conditions. While in the local conditions, the numbers of mismatches decrease very significantly (by 62% and by 59% in the red and blue conditions respectively), the reduction in the global condition is by 33%. Compared to the CIELAB results, in all conditions the reduction was similar (54%, 53%, and 63% in global, red and blue).

The red condition. In the red condition, the categorization bias appears to be most influential, whereas the lightness-saturation bias does not alter the picture. This is consistent with our result for the CIELAB model. The optimal categorization solution, figure 9(b) of this document for CIEDE2000 and figure 2 in the main text for CIELAB, is unique. The two solutions are similar to each other.

The blue condition. In the blue condition, the lightness-saturation bias is more important, but adding the categorization bias helps improve the model even further. Again, this is consistent with our result for the CIELAB model.

The optimal categorization for CIELAB (figure 2 of the main text) is unique, and the optimal categorization for CIEDE2000 (figure 9(c) of this document) consists of three very similar ways of splitting the colors, all very similar to the one depicted in figure figure 9(c).

The global condition. In the global condition, both categorization and lightness-saturation biases appear to play a role. The power of the modified model, however, is more significant when used in combination with the CIELAB distance formula (where it reduces the number of mismatches from 13 to 5), compared with the CIEDE2000 formula, where the reduction is less significant (from 12 to 8). In the latter case, we observe many different optimal categorization solutions, all resulting in 8 mismatches. One of these solutions is shown in figure 9(a).

References

[1] Berlin, B., & Kay, P. (1969). *Basic color terms: Their universality and evolution.* Berkeley: University of California Press.

[2] Bimler, D., Kirkland, K. & Pichler, S. (2004). Escher in color space: Individual-differences multidimensional scaling of color dissimilarities collected with a gestalt formation task. Behavior Research Methods, Instruments, & Computers 2004, 36 (1), 69-76

[3] Boynton, R. M. & Olson, C. (1987). Locating basic colors in the OSA space. COLOR Research and Application, 12, 94–105.

[4] Burton, M.L. & S.B. Nerlove. 1976. Balanced Designs for Triads Tests: Two Examples from English. Social Science Research 5, 247–267.

[5] Coombs, C. H. (1964). *A Theory of Data.* Wiley, New York.

[6] D'Andrade, R. G. (2003). English color naming data for 424 OSA color tiles. Unpublished research report. University of California, San Diego. Department of Anthropology.

[7] CIE94: DeCusatis, C. (1997). *Handbook of Applied Photometry*, Aip Press Series, Optical Society of America. Springer publishers. Page 370, Eq. 10.54.

[8] CIE94: DeCusatis, C. (1997). *Handbook of Applied Photometry*, Aip Press Series, Optical Society of America. Springer publishers. Page 372, Eq. 10.66.

[9] Indow, T. (1988). Multidimensional studies of Munsell color solid. Psychological Review, 95,456-470.

[10] Jameson, K. A., Highnote, S. M., & Wasserman, L. M. (2001). Richer color experience in observers with multiple photopigment opsin genes. Psychonomic Bulletin & Review, 8, 244-261.

[11] Luo, M.R. and Cui, G. and Rigg, B. (2001) The development of the CIE 2000 colour-difference formula: CIEDE2000, Color Research & Application 26, 340–350.

[12] Luo, M. R. (2006). Colour difference formulae: past, present and future. (pp. 145-150). In the Proceedings of the ISCC/CIE Expert Symposium 2006. "75 Years of the CIE Standard Colorimetric Observer", 16-17 May 2006. NRC, Ottawa, Ontario, Canada. Publ. CIE x030:2006.

[13] Moore, C. C., Romney, A. K., & Hsia, T.-L. (2002). Cultural, gender, and individual differences in perceptual and semantic structures of basic colors in Chinese and English. Journal of Cognition and Culture, 2, 1–28.

[14] Romney, A. K., Boyd, J. P., Moore, C. C., Batchelder, W. H. & Brazill, T. J. (1996) Proc. Natl. Acad. Sci. USA 93, 4699–4705.

[15] Paramei, G.V. (2005) Singing the Russian blues: An argument for culturally basic color terms. Cross-Cultural Research 39, 10–38.

[16] Romney, A.K., Weller, S.C. & Batchelder, W.H. (1986). Culture as consensus: A Theory of culture and informant accuracy. American Anthroplogist 99,313-338

[17] Sayim, B., Jameson, K. A., Alvarado, N. and Szeszel, M. K. (2005). Semantic and Perceptual Representations of Color: Evidence of a Shared Color–Naming Function. The Journal of Cognition & Culture, 5(3/4), 427–486.

[18] Sharma, G. and Wu, W. and Dalal, E.N. (2005) The CIEDE2000 color-difference formula: Implementation notes, supplementary test data, and mathematical observations, Color Research & Application 30, 21–30.

[19] Wang, H., Cui, G., Luo, M. R., & Xu, H. (2011). Evaluation of colour-difference formulae for different colour-difference magnitudes. Color Research and Application, 37(5), 316–325.

[20] Weller, S. C. & Romney, A. K. (1988). Systematic Data Collection. Qualitative Research Methods, Volume 10. Sage Publications: London.
